# SLAM family member 8 is expressed in and enhances the growth of anaplastic large cell lymphoma

**DOI:** 10.1038/s41598-020-59530-1

**Published:** 2020-02-13

**Authors:** Akihiko Sugimoto, Tatsuki R. Kataoka, Hiroaki Ito, Kyohei Kitamura, Narumi Saito, Masahiro Hirata, Chiyuki Ueshima, Yusuke Takei, Koki Moriyoshi, Yasuyuki Otsuka, Momoko Nishikori, Akifumi Takaori-Kondo, Hironori Haga

**Affiliations:** 1 Department of Diagnostic Pathology, Kyoto University Hospital, Kyoto, Japan; 2 Department of Diagnostic Pathology, Saiseikai-Noe Hospital, Osaka, Japan; 3 Department of Diagnostic Pathology, Kyoto Medical Center, Kyoto, Japan; 4 Department of Hematology/Oncology, Kyoto University Hospital, Kyoto, Japan

**Keywords:** T-cell lymphoma, Cell growth, Oncogenesis

## Abstract

Signaling lymphocytic activation molecule family member 8 (SLAMF8) / B-lymphocyte activator macrophage expressed/CD353 is a member of the CD2 family. SLAMF8 suppresses macrophage function but enhances the growth of neoplastic mast cells via SHP-2. In this study, we found that some anaplastic large cell lymphoma (ALCL) samples were immunohistochemically positive for SLAMF8. However, we found no significant differences between SLAMF8-positive and SLAMF8-negative ALCL samples with respect to age, gender, site, or prognosis. We also identified SLAMF8 expression in ALCL cell lines, Karpas299, and SU-DHL-1. SLAMF8 knockdown decreased the activation of SHP-2 and the growth of these cell lines, and increased the apoptosis of these cell lines. In addition, we observed the interaction between SLAMF8 and SHP-2 in these cell lines using the DuoLink *in situ* kit. Taken together, these results suggest that SLAMF8 may enhance the growth of ALCL via SHP-2 interaction.

## Introduction

The signaling lymphocytic activation molecule (SLAM) family of receptors is expressed by and regulates the functions of immune cells^1,2^. SLAM family member 8 (SLAMF8)/B-lymphocyte activator macrophage expressed/CD353 is a cell-surface protein and a member of the CD2 family^1,2^. However, its ligand has not yet been identified. SLAMF8 is a potent marker for tumor infiltrating T cells. SLAMF8 are abundantly expressed in T cells infiltrating in pediatric cancers and Epstein-Barr virus-positive gastric cancers, and thought to be a marker for potential immunotherapy targets^3,4^. In addition, SLAMF8 is expressed by and suppresses macrophage the function of human macrophage^5,6^. SLAMF8 is also expressed by and enhances the growth of human neoplastic mast cells^7^. SLAM family signaling is generally mediated by SLAM-associated protein (SAP) or Ewing’s sarcoma-associated transcript 2 (EAT-2) in lymphocytes and macrophage cells^1,2^, although SLAMF8 signaling is mediated by the Src homology region 2 domain-containing phosphatase-2 (SHP-2) in human mast cells^7^. To the best of our knowledge, no studies have explored the expression of SLAMF8 in anaplastic large cell lymphoma (ALCL).

ALCL cells are typically large, have abundant cytoplasm and horseshoe-shaped nuclei, and are positive for CD30 on the cell membrane and in the Golgi region^8^. The expression of anaplastic lymphoma kinase (ALK) is clinically important, and the prognosis of ALK-positive ALCL is favorable compared to ALK-negative ALCL. There are three subtypes of ALCL: ALK-positive, ALK-negative, and primary cutaneous ALCL. In this study, we analyzed the expression and function of SLAMF8 in ALCL clinical samples and cell lines.

## Results

### Human ALCLs express SLAMF8 mRNA and protein

**Figure 1:**
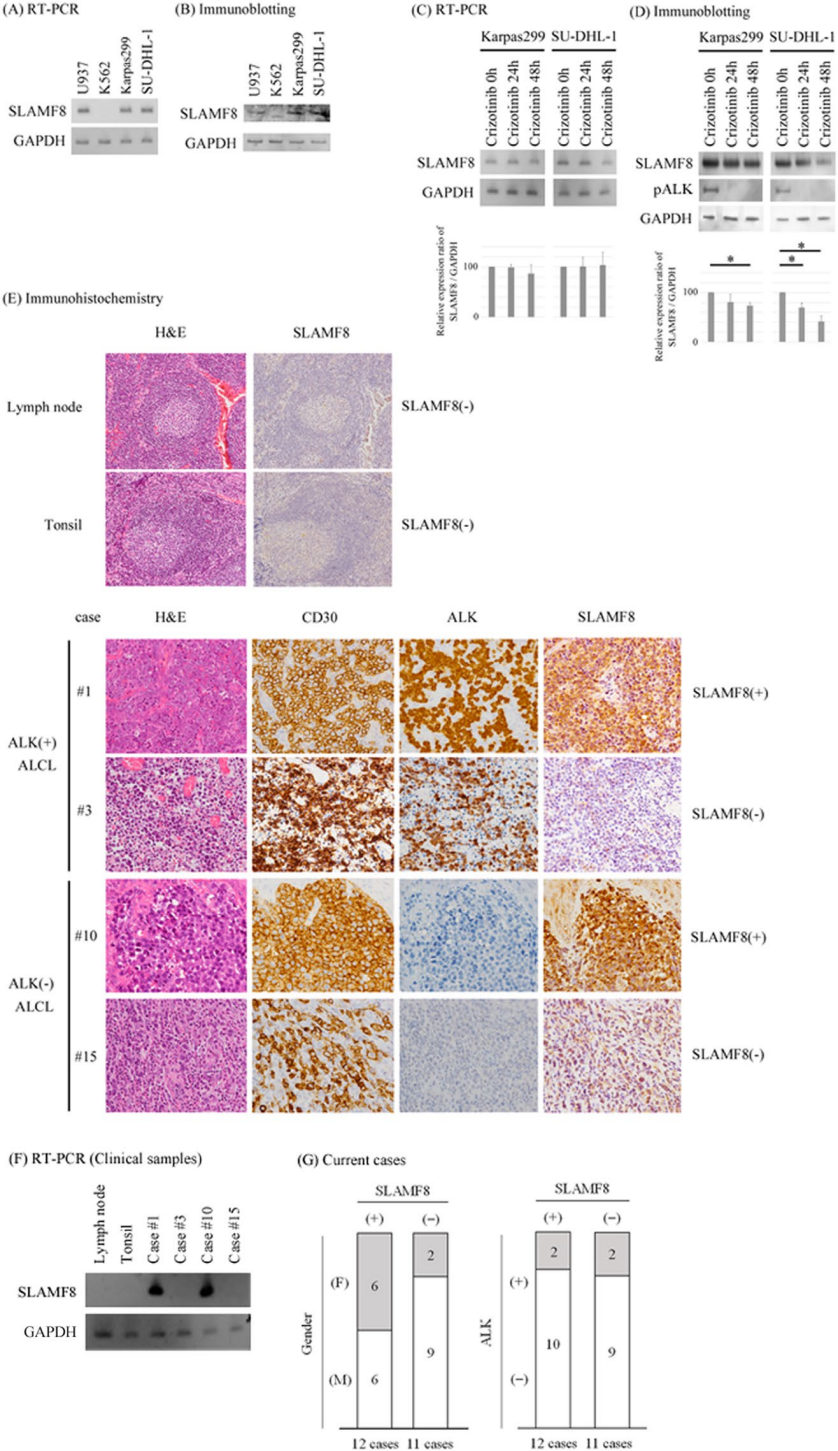
SLAMF8 is expressed in human ALCL cell lines Karpas299 and SU-DHL-1 and in human ALCL pathological samples. (**A**) RT-PCR for cell lines. Cropped gels are shown and the gels were run under the same conditions. U937 is used as a positive control, and K562 is used as a negative control. (**B**) Immunoblotting for cell lines. Cropped blots are shown and the gels were run under the same conditions. U937 is used as a positive control, and K562 is used as a negative control. (**C**) RT-PCR for crizotinib-treated ALCL cell lines. Cropped gels are shown and the gels were run under the same conditions. The band intensities were analyzed and the relative SLAMF8/GAPDH ratio was calculated. ^*^P < 0.05, compared to ALCL cells treated with crizotinib 0 h. (**D**) Immunoblotting for crizotinib-treated ALCL cell lines. Cropped blots are shown and the gels were run under the same conditions. The band intensities were analyzed and the relative SLAMF8/GAPDH ratio was calculated. ^*^P < 0.05, compared to ALCL cells treated with crizotinib 0 h. (**E**) Immunohistochemistry. Representative cases are shown. H&E, hematoxylin and eosin. (**F**) RT-PCR for pathological specimens. Total mRNAs from macro-dissected samples were used. Cropped gels are shown and the gels were run under the same conditions. (**G**) The association between SLAMF8 protein expression and clinical parameters, gender, and ALK expression.

We examined the expression of SLAMF8 mRNA and protein in the human ALCL cell lines SU-DHL-1 and Karpas299 using RT-PCR and immunoblotting, respectively. Both cell lines expressed SLAMF8 mRNA and protein (Fig. 1A,B). Both cell lines showed ALK translocation^9,10^ and we treated these with an ALK inhibitor, crizotinib^11^. The administration of crizotinib did not significantly affect the expression of SLAMF8 mRNA in either cell line (Fig. 1C) but significantly decreased the expression of SLAMF8 protein in both cell lines (Fig. 1D), after we confirmed the inhibitory effect of crizotinib on the phosphorylations of ALK in both cell lines (Fig. 1D).

Next, we examined the expression of SLAMF8 protein in human pathological specimens of non-neoplastic lymph nodes, non-neoplastic tonsils, and ALCL by immunohistochemistry. Lymphocytes, including non-neoplastic lymph node and non-neoplastic tonsil specimens, were negative for SLAMF8 protein and mRNA (Fig. 1E,F). Overall, 12 of 23 ALCL cases (52.2%) were positive for SLAMF8 protein and mRNA (Table 1, Fig. 1E,F). SLAMF8 expression was not associated with age, gender, prognoses, ALK status, or lesion site (Table 2 and Fig. 1G).

**Table 1:** Clinical characteristics of ALCL patients.

Case	Age (y.o.)	Gender	Site	Classification	Ann Arbor staging	CD30	ALK	SLAMF8
1	24	F	Lymph node (inguinal)	systemic	IV	positive	positive	positive
2	41	F	Skin (lower limb)	systemic	IV	positive	positive	positive
3	9	F	Urinary bladder	systemic	IV	positive	positive	negative
4	63	M	Bone (limb)	systemic	IV	positive	positive	negative
5	14	M	Bone (vertebra)	systemic	IE	positive	negative	positive
6	39	M	Skin (back)	systemic	I	positive	negative	positive
7	40	F	Bone (upper limb)	systemic	IE	positive	negative	positive
8	55	M	Stomach	systemic	IE	positive	negative	positive
9	56	F	Lymph node (neck)	systemic	IE	positive	negative	positive
10	65	M	Skin (lower limb)	cutaneous	I	positive	negative	positive
11	70	M	Bone (ileic)	systemic	IV	positive	negative	positive
12	75	F	Soft tissue (buttock)	systemic	IE	positive	negative	positive
13	75	M	Lung	systemic	IV	positive	negative	positive
14	93	F	Skin (foot)	cutaneous	I	positive	negative	positive
15	1	F	Skin (upper limb)	cutaneous	I	positive	negative	negative
16	31	M	Lymph node (inguinal)	cutaneous	I	positive	negative	negative
17	40	M	Skin (chest wall)	cutaneous	I	positive	negative	negative
18	63	M	Soft tissue (inguinal)	systemic	IV	positive	negative	negative
19	66	M	Lymph node (neck)	systemic	IV	positive	negative	negative
20	68	M	Lymph node (inguinal)	systemic	II	positive	negative	negative
21	68	M	Skin (scalp)	cutaneous	I	positive	negative	negative
22	71	M	Lymph node (neck)	systemic	II	positive	negative	negative
23	76	M	Skin (upper limb)	cutaneous	I	positive	negative	negative

CD30, ALK, and SLAMF8 were evaluated by immunohistochemistry.

**Table 2:** Summary of ALCL pathological results.

	SLAMF8-positive	SLAMF8-negative	Total
Case number (cases)	12	11	23
Average age (y.o.)	53.9 (14–93)	50.5 (1–76)	52.3 (1–93)
Gender (F:M)	6:6	2:9	8:15
CD30	12/12 (100%)	11/11 (100%)	23/23 (100%)
ALK	2/12 (16.7%)	2/11 (18.2%)	4/23 (17.4%)
SLAMF8	12/12 (100%)	0/11 (0%)	12/23 (52.2%)

CD30, ALK, and SLAMF8 were evaluated by immunohistochemistry.

### SLAMF8 knockdown decreases the growth of and increases the apoptosis of ALK-positive ALCL cells

**Figure 2:**
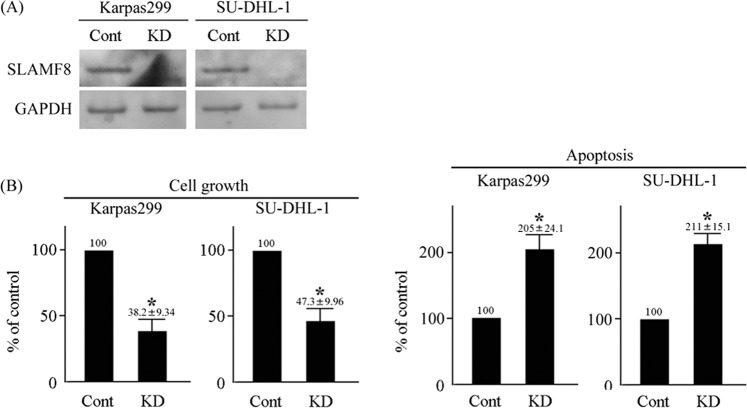
SLAMF8 knockdown decreased the growth of ALK-positive ALCL cell lines. (**A**) SLAMF8 knockdown in human ALCL cell lines. Cropped blots are shown and the gels were run under the same conditions. (**B**) The effects of SLAMF8 knockdown on cell growth and apoptosis in human ALCL cell lines. CCK-8 assay and Annexin V-FITC staining/propidium iodine incorporation (n = 3, respectively). ^*^P < 0.05, compared to control cells.

The ligand for SLAMF8 has not yet been identified. Therefore, we knocked-down SLAMF8 in human ALCL cell lines to explore the function of this receptor using shRNA targeting human SLAMF8 (Fig. 2A). We assessed the effect on the growth and apoptosis of ALCL cell lines after knockdown of SLAMF8. SLAMF8 knockdown decreased the growth and increased the apoptosis of both ALCL cell lines (Fig. 2B).

### SHP-2 mediates the growth enhancing activity of and interacts with SLAMF8 in ALK-positive ALCL cells

**Figure 3:**
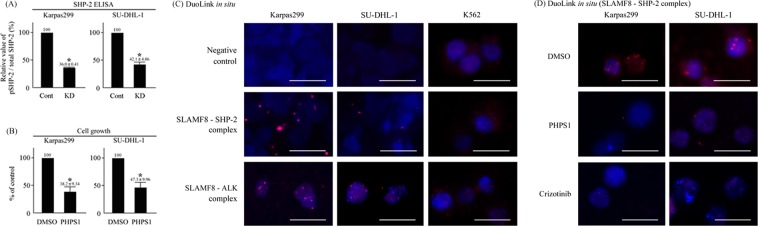
SHP-2 mediates the growth enhancing activity of and interacts with SLAMF8 in ALK-positive ALCL cells. (**A**) The effects of SLAMF8 knockdown on SHP-2 activation in human ALCL cell lines. SHP-2 ELISA (n = 3). ^*^P < 0.05, compared to control cells. (**B**) The effects of the SHP-2 inhibitor PHPS1 on the cell growth of human ALCL cell lines. CCK-8 assay (n = 3). ^*^P < 0.05, compared to control cells. (**C**) Interaction between SLAMF8 and SHP-2 or ALK in human cell lines. The DuoLink *in situ* kit. Red dots indicate the interaction between SLAMF8 and SHP-2 or ALK. Bars; 10 μM. (**D**) Interaction between SLAMF8 and SHP-2 in ALK-positive ALCL cell lines treated with crizotinib or PHPS1. Red dots indicate the interaction between SLAMF8 and SHP-2. Bars; 10 μM.

We assessed the status of signaling molecules in ALCL cell lines using an ELISA kit. SHP-2 plays a central role in the growth of ALCL cells^12^, and we previously reported that SHP-2 is important for SLAMF8-associated neoplastic mast cell growth^7^. Therefore, we assessed the status of SHP-2 in ALCL cells with SLAMF8 knockdown. The ELISA assay showed that SLAMF8 knockdown in ALCL cells decreased the activation of SHP-2 (Fig. 3A). We also confirmed that the SHP-2 inhibitor PHPS1^13^ suppressed the growth of ALCL cells at levels equivalent to those seen in the SLAMF8 knockdown cells (Fig. 3B).

Next, we investigated the potential interactions between SLAMF8 and SHP-2 in ALCL cell lines using the DuoLink *in situ* kit^14^. SLAMF8 was shown to interact with both SHP-2 and ALK in both ALCL cell lines, but not in K562 cells (Fig. 3C). We could not identify the SLAMF8–SHP-2 complex in ALCL cell lines when treated with crizotinib or PHPS1 (Fig. 3D).

## Discussion

SLAMF8 suppresses the functions of human macrophages^5,6^ but has an enhancing effect in human ALK-positive ALCL cell lines and in human KIT-mutated neoplastic mast cell lines^7^. We previously hypothesized that this difference was due to the differential expression of adaptor proteins between human macrophages and KIT-mutated neoplastic mast cells; specifically, human macrophages express the adaptor proteins, SAP and EAT-2, which interacting with SLAMF8^15^. We are suspecting that these adaptor proteins interfere with the interaction between SHP-2 and SLAMF8 in human macrophage, and the absent expression of SAP and EAT-2 facilitate the interaction between SHP-2 and SLAMF8 in human neoplastic mast cells^7,16^. In this study, we observed SAP-2 and EAT-2 expression in ALK-positive ALCL cell lines (data not shown). Other mechanisms in ALCL cells may be possible but have not yet been investigated.

SHP-2 is constitutively phosphorylated by ALK in ALCL^12^ and by mutated KIT in neoplastic mast cells^17^ but not in non-neoplastic macrophages. Administration of an ALK inhibitor crizotinib should limit the phosphorylation of SHP-2, as is the case of the administration with a SHP-2 inhibitor PHPS1, in Karpas299 and SU-DHL-1 cells, and we found that administration of crizotinib or PHPS1 disrupted the interaction between SLAMF8 and SHP-2 in both cell lines. This suggests that phosphorylation of SHP-2 is required for the interaction between SLAMF8 and SHP-2 and the followed enhanced effects of SLAMF8 in ALK-positive ALCL cells. The administration of crizotinib decreased the SLAMF8 protein level but not SLAMF8 mRNA level in both cell lines. These observations might indicate that SLAMF8 protein is degraded when the complex with phospho-SHP-2 is not formed, though the further examinations should be performed to support this hypothesis. Here, we could not detect the expression of SLAMF8 protein and mRNA on lymphocytes including non-neoplastic lymph nodes or tonsils, although some reports support the expression of SLAMF8 on tumor infiltrating lymphocytes (TILs)^3,4^. SHP-2 may be phosphorylated in SLAMF8-positive TILs, although not phosphorylated in SLAMF8-negative lymphocytes including non-neoplastic lymph nodes or tonsils, as is the case of non-neoplastic macrophages.

We also found the SLAMF8 expression in ALK-negative ALCL specimens. STAT3 is known to be activated in both ALK-positive and ALK-negative ALCLs^18^, and we found that the administration with a STAT3 inhibitor Stattics declined the SLAMF8 expression protein levels in the ALK-positive ALCL cell lines (data not shown). These indicate the possibility of the involvement of STAT3 in the SLAMF8 expression in ALK-negative ALCL. The detailed mechanism should be studied in the future using ALK-negative ALCL cell lines.

Taken together, SLAMF8 is expressed in and enhances the cell growth of ALCL cells via SHP-2 activation, and that SLAMF8 interacts with activated SHP-2 and activated ALK proteins in ALK-positive ALCL cells.

## Methods

### Patients

Histological specimens diagnosed as ALCL were obtained from Kyoto University Hospital (Sakyo-ku, Kyoto, Japan) and Kyoto Medical Center (Fushimi-ku, Kyoto, Japan). Patients over 18 years old or parent(s) of patients under 18 years old from a parent attending Kyoto University Hospital signed the “Kyoto University Hospital Informed Consent Form for the Non-therapeutic Use of Histopathological Materials,” and the signed forms were uploaded into all electronic health records. We also obtained informed consent and written permission from patients at the Kyoto Medical Center and the signed forms were uploaded into all electronic health records. Table 1 summarizes patient clinical characteristics. Clinical data and samples were used with the approval of the Institutional Review Board of Kyoto University Hospital.

### Cells

ALK-positive ALCL cell lines, SU-DHL-1 and Karpas299^9,10^ were provided by Drs Y. Matsuo (Fujisaki Cell Center, Okayama, Japan) and H. Drexler (Deutsche Sammlung von Mikroorganismen und Zellkulturen [DSMZ], Braunschweig, Germany). K562 (negative control for SLAMF8) and U937 (positive control for SLAMF8) cells were purchased from the American Type Culture Collection (ATCC, Manassas, VA, USA)^7^. The cells were cultured in RPMI1640 + 10% fetal calf serum (FCS).

### Immunohistochemistry

Tissue sections were deparaffinized, activated at 95 °C in Dako target retrieval agent (pH 9, DakoCytomation, Glostrup, Denmark) for 30 min, and pretreated with 3% H_2_O_2_ for 5 min. An anti-SLAMF8 antibody (×200, rabbit polyclonal, bs-2473R; Bioss, Woburn, MA, USA), an anti-ALK antibody (mouse monoclonal antibody, 5A4, Leica Microsystems, Wetzlar, Germany), or an anti-CD30 antibody mouse monoclonal antibody (Ber-H2, Roche Diagnostics, Mannheim, Germany) was added and incubated at room temperature for 1 h. For staining, the EnVision kit was used according to the manufacturer’s instructions (HRP/DAB; DAKO). We defined positivity as when more than 50% of tumor cells were stained in their membranes and cytoplasms.

### RT-PCR

To assess the SLAMF8 mRNA expression in cell lines, 1–3 × 10^5^ ALCL cells in total were processed with TRIzol reagent (Invitrogen, Carlsbad, CA, USA) and maintained at −20 °C overnight. In a separate experiment, ALCL cells were cultured for 0 h, 24 h, or 48 h at 1–3 × 10^4^ cells/100 µL/well in the ALK inhibitor crizotinib (Sigma-Aldrich, St. Louis, MO, USA) at 10 nM^11^ and processed. Total RNA was extracted using the PureLinkTM RNA Mini Kit (12183018 A; Life Technologies, Tokyo, Japan). Complementary DNA was synthesized from the collected RNA using a Reverse Transcription System (Promega, Tokyo, Japan). Both kits were used according to the manufacturer’s instructions. Then we performed PCR using the primers 5′-CCCAACATCAGCGAAATAACC-3′ and 5′-GAAGAGAGTAACCAGCATCAG-3′^7^. PCR was performed as follows: 94 °C at 5 min, followed by 28 cycles of 94 °C for 30 s, 61 °C for 30 s, and 72 °C for 30 s, and a final extension at 72 °C for 10 min. The PCR products were subjected to electrophoresis and scanned using a Light-Capture II (ATTO, Tokyo, Japan). To quantify the band intensities, the scanned data were analyzed using the CS Analyzer (ATTO).

To assess the SLAMF8 mRNA expression in pathological specimens, total RNA was extracted from macro-dissected specimens using the RNeasy FFPE Kit (#73504; QIAGEN, Hilden, Germany) according to the manufacturer’s instructions. Complementary DNA was synthesized from the collected RNA using a Reverse Transcription System (Promega). We performed PCR using the above-mentioned primers. PCR was performed as follows: 94 °C at 5 min, followed by 58 cycles of 94 °C for 30 s, 59 °C for 30 s, and 72 °C for 30 s, and a final extension at 72 °C for 10 min. The PCR products were subjected to electrophoresis and scanned using a Light-Capture II (ATTO).

### Immunoblotting

A total of 1–3 × 10^4^ cells were lysed using CellLytic (Sigma-Aldrich). We used an iBlot kit (Invitrogen) to perform immunoblotting. The antibodies used to detect proteins were as follows: Anti-SLAMF8 antibody (rabbit polyclonal, PAB8582; Abnova, Heidelberg, Germany), anti-phospho-ALK (Tyr 1096) (rabbit polyclonal, #4143; Cell Signaling Technology, Beverly, MA, USA) and anti-GAPDH antibody (rabbit monoclonal [D16H11], #5174; Cell Signaling Technology). The blotting images were scanned using a Light-Capture II (ATTO) (Figs. 1 suppl and 2 suppl). To quantify the band’s intensities, the scanned data were analyzed using the CS Analyzer (ATTO).

### SLAMF8 knockdown

SU-DHL-1 or Karpas299 cells were infected with BLAME short hairpin RNA (shRNA) (h) lentiviral particles (sc-60273-V; Santa Cruz Biotechnology, San Diego, CA, USA) or control shRNA lentiviral particles-A (sc-108080; Santa Cruz Biotechnology) according to the manufacturer’s instructions. We termed the infected cells “SLAMF8-KD” and the control cells “Control.”

### Cell growth assay

Control and SLAMF8-KD ALCL cells were cultured for 24 h at 1–3 × 10^4^ cells/100 µL/well in RPMI1640 + 10% FCS. In a separate experiment, ALCL cells were cultured for 24 h at 1–3 × 10^4^ cells/100 µL/well in the presence of dimethyl sulfoxide (DMSO) or the SHP-2 specific inhibitor PHPS1 (Santa Cruz Biotechnology) at 10 μM.^9^ The Cell Counting Kit-8 (Dojindo, Kumamoto, Japan) was used to evaluate growth by measuring absorbance at 450 nm. The relative absorbance was calculated based on the absorbance of control cells or DMSO. Three individual experiments were performed.

### Apoptosis assay

We analyzed the apoptosis of ALCL cell lines using Annexin V-FITC Apoptosis Kit Plus (Biovision, Milpitas, CA, USA). Control and SLAMF8-KD ALCL cells were cultured for 24 h at 1–3 × 10^4^ cells/100 µL/well in RPMI1640 + 10% FCS. The cells were analyzed according to the manufacturer’s instructions. The FITC-positive / PI-negative cells were considered apoptotic cells. Three individual experiments were performed.

### Assessment of phospho-SHP-2 levels

We measured the ratios of phospho-SHP-2/total SHP-2 using a human/mouse/rat phospho-SHP2 (Y542) and total SHP2 enzyme-linked immunosorbent assay (ELISA) kit (RayBiotech, Norcross, GA, USA). Control and SLAMF8-KD ALCL cells were cultured for 1 h at 1–3 × 10^4^ cells/100 µL RPMI1640 containing 10% FCS. After centrifugation, cell pellets were processed and subjected to ELISA according to the manufacturer’s instructions. Three individual experiments were performed.

### Co-localization of SLAMF8 with SHP-2 or ALK

The DuoLink *in situ* kit (Sigma-Aldrich) was used to evaluate the co-localization of SLAMF8 with SHP-2 or ALK according to the manufacturer’s instructions.^10^ We used an anti-SHP-2 antibody (goat polyclonal antibody, ab9214; Abcam, Cambridge, UK), an anti-ALK antibody (5A4), and an anti-SLAMF8 antibody (rabbit polyclonal antibody, bs-2473R; Bioss). We collected and centrifuged untreated Karpas299, untreated SU-DHL-1, untreated K562, DMSO-treated (24 h) Karpas299, DMSO-treated (24 h) SU-DHL-1, crizotinib-treated (24 h) Karpas299, and crizotinib-treated (24 h) SU-DHL-1 cells and resuspended the cell pellets in 20% (v/v) buffered formalin (pH 7.0) followed by centrifugation for 5 min at 1,500 rpm. Then the cell pellets were dehydrated and embedded in paraffin and the paraffin blocks were cut into 3–4 μm thick sections prior to hematoxylin and eosin (H&E) staining and DuoLink evaluation. The slides were mounted with fluoroshield mounting medium with 4′,6-diamidino-2-phenylindole (DAPI). Sections were imaged with a BX63 microscope (Olympus, Tokyo, Japan) equipped with an ORCA Flash 2.8 digital camera (Hamamatsu Photonics, Shizuoka, Japan).

### Statistical analyses

Data are expressed as means ± standard errors of the mean (SEs). Differences between groups were examined for statistical significance using Student’s *t*-test (Excel, Microsoft, Redmond, WA, USA). A *P* value less than 0.05 indicated statistical significance.

## Supplementary information

Figure 1 suppl dataset.

Figure 2 suppl dataset.

## Data Availability

All data generated or analysed during this study are included in this published article.
