# Supplementary material for: SLAM family member 8 is expressed in and enhances the growth of anaplastic large cell lymphoma
Source: Sci Rep. 2020 Feb 13;10:2505. doi: 10.1038/s41598-020-59530-1 (PMC7018816; doi:10.1038/s41598-020-59530-1)
Supplement: Supplementary file 1 — Figure 1 suppl dataset. [file 41598_2020_59530_MOESM1_ESM.pdf]

SREP-18-43975C

SLAM family member 8 is expressed in and enhances the growth of anaplastic large cell lymphoma.

Akihiko Sugimoto,[a] *Tatsuki R. Kataoka,[a] Hiroaki Ito,[a] Kyohei Kitamura,[a] Narumi Saito,[a] Masahiro Hirata,[a] Chiyuki Ueshima,[a] Yusuke Takei,[a,b] Koki Moriyoshi,[a,c] Yasuyuki Otsuka,[d] Momoko Nishikori,[d] Akifumi Takaori-Kondo,[d] and Hironori Haga.[a]

[a] Department of Diagnostic Pathology and [d] Department of Hematology/Oncology, Kyoto University Hospital, Kyoto, Japan

[b] Department of Diagnostic Pathology, Saiseikai-Noe Hospital, Osaka , Japan

[c] Department of Diagnostic Pathology, Kyoto Medical Center, Kyoto, Japan

Figure 1 suppl files

(A) RT-PCR

SLAMF8

Lane 1, Marker; Lane 2, U937; Lane 3, K562; Lane 4, Karpas299; Lane 5, SU-DHL-1.

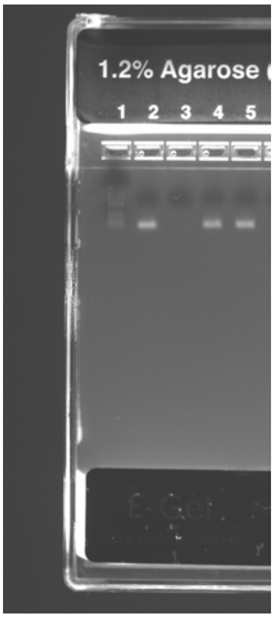

GAPDH

Lane 7, Marker; Lane 8, U937; Lane 9, K562; Lane 10, Karpas299; Lane 11, SU-DHL-1.

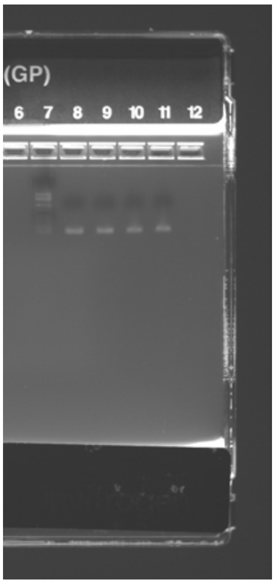

(B) Immunoblotting

SLAMF8

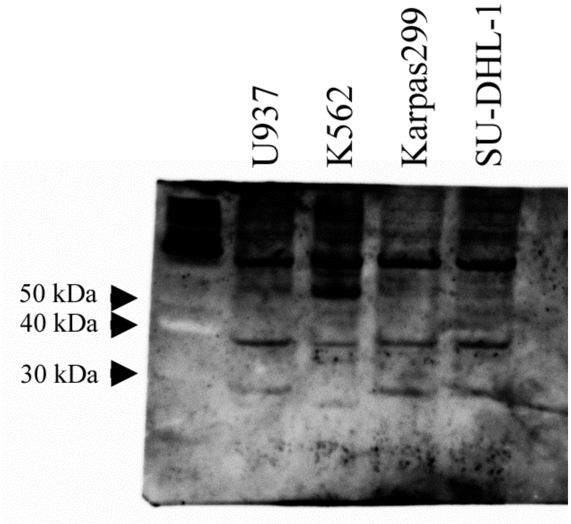

GAPDH

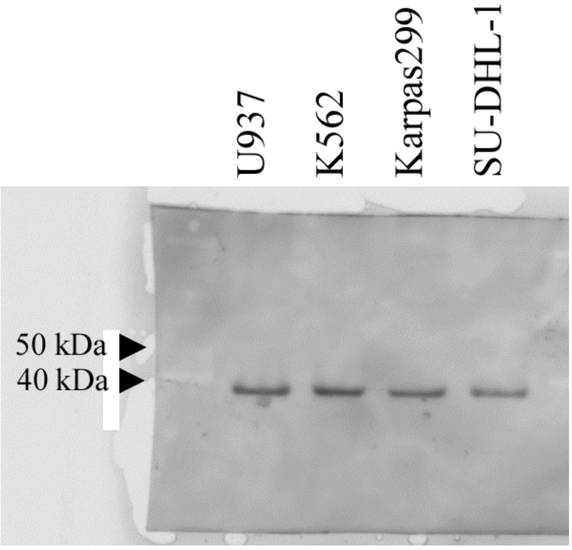

(C) RT-PCR

SLAMF8

Lane 1, Marker; Lane 2, Marker; Lane 3, Blank; Lane 4, Karpas299 untreated; Lane 5, Karpas299 crizotinib-treated for 24 h; Lane 6, Karpas299 crizotinib-treated for 48 h; Lane 7, Blank; Lane 8, SU-DHL-1 untreated; Lane 9, SU-DHL-1 crizotinib-treated for 24 h; Lane 10, SU-DHL-1 crizotinib-treated for 48 h; Lane 11, Blank; Lane 12, Marker.

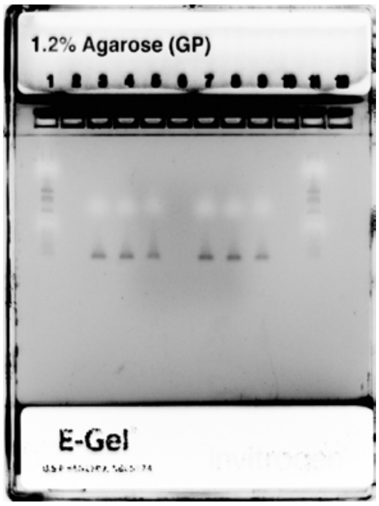

GAPDH

Lane 1, Marker; Lane 2, Marker; Lane 3, Blank; Lane 4, Karpas299 untreated; Lane 5, Karpas299 crizotinib-treated for 24 h; Lane 6, Karpas299 crizotinib-treated for 48 h; Lane 7, Blank; Lane 8, SU-DHL-1 untreated; Lane 9, SU-DHL-1 crizotinib-treated for 24 h; Lane 10, SU-DHL-1 crizotinib-treated for 48 h; Lane 11, Blank; Lane 12, Marker.

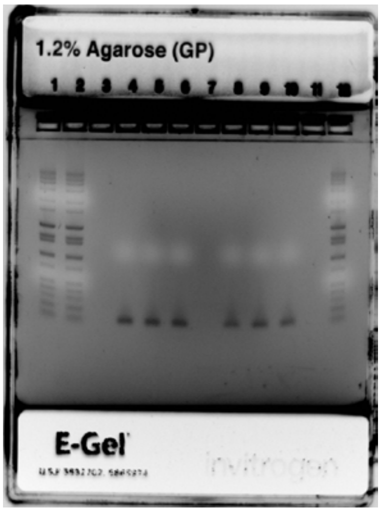

(D) Immunoblotting

SLAMF8

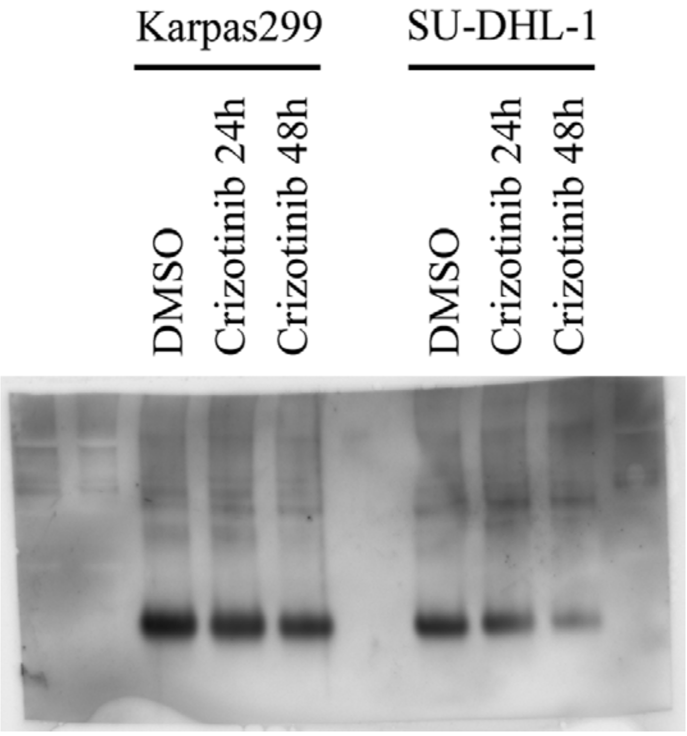

pALK

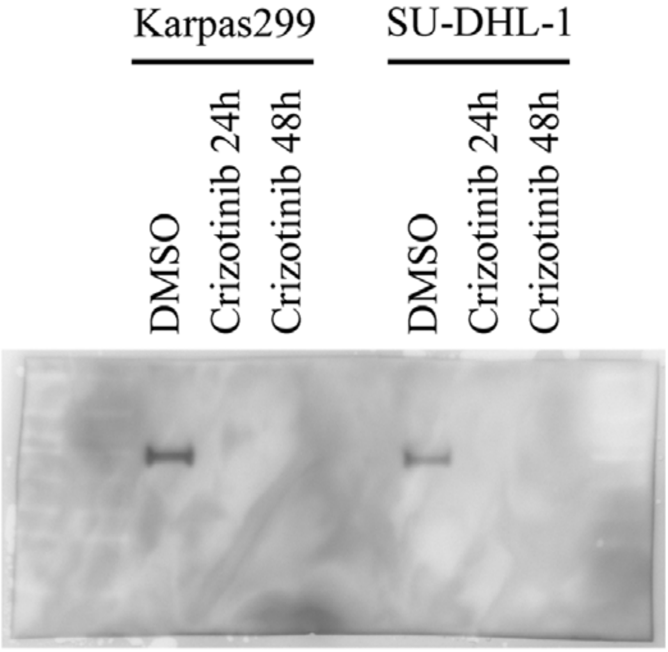

GAPDH

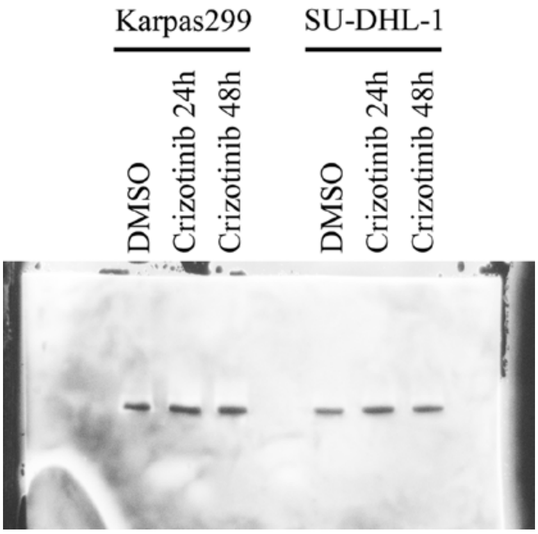
Karpas299
SU-DHL-1
DMSO
Crizotinib 24h
Crizotinib 48h
DMSO
Crizotinib 24h
Crizotinib 48h

(F) RT-PCR

SLAMF8

Lane 1, Marker; Lane 2, Lymph node; Lane 3, Tonsil; Lane 4, Case #1; Lane 5, Case #3; Lane 6, Case #10; Lane 7, Case #15; Lane 8, Marker.

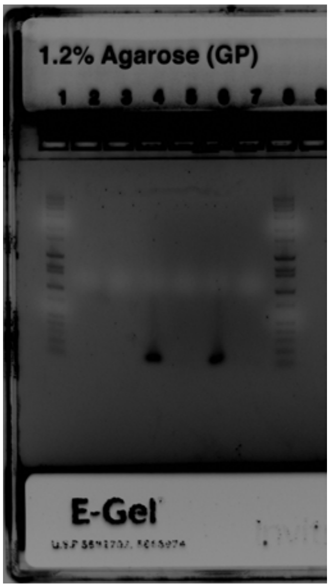

GAPDH

Lane 5, Marker; Lane 6, Lymph node; Lane 7, Tonsil; Lane 8, Case #1; Lane 9, Case #3; Lane 10, Case #10; Lane 11, Case #15; Lane 12, Marker.

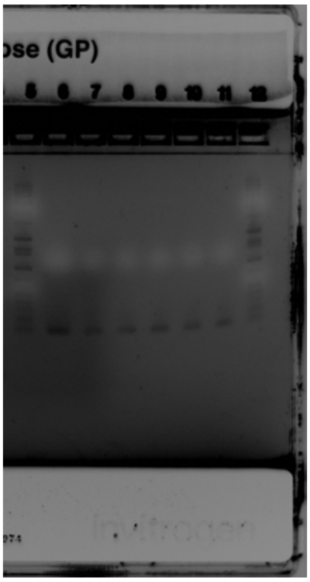

END
