# Supplementary material for: SLAM family member 8 is expressed in and enhances the growth of anaplastic large cell lymphoma
Source: Sci Rep. 2020 Feb 13;10:2505. doi: 10.1038/s41598-020-59530-1 (PMC7018816; doi:10.1038/s41598-020-59530-1)

SREP-18-43975C

SLAM family member 8 is expressed in and enhances the growth of anaplastic large cell lymphoma.

Akihiko Sugimoto,[a] *Tatsuki R. Kataoka,[a] Hiroaki Ito,[a] Kyohei Kitamura,[a] Narumi Saito,[a] Masahiro Hirata,[a] Chiyuki Ueshima,[a] Yusuke Takei,[a,b] Koki Moriyoshi,[a,c] Yasuyuki Otsuka,[d] Momoko Nishikori,[d] Akifumi Takaori-Kondo,[d] and Hironori Haga.[a]

[a] Department of Diagnostic Pathology and [d] Department of Hematology/Oncology, Kyoto University Hospital, Kyoto, Japan
[b] Department of Diagnostic Pathology, Saiseikai-Noe Hospital, Osaka , Japan
[c] Department of Diagnostic Pathology, Kyoto Medical Center, Kyoto, Japan

Figure 2 suppl files

Immunoblotting; SLAMF8 (upper) & GAPDH (bottom)

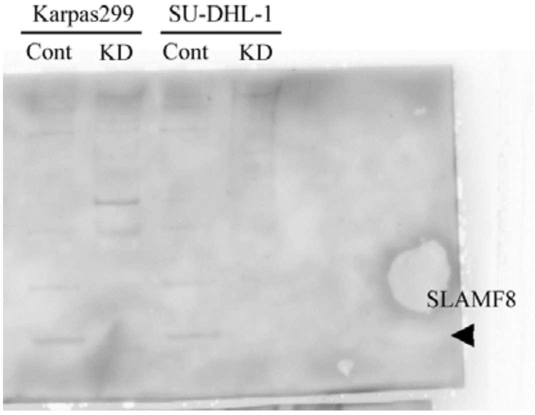

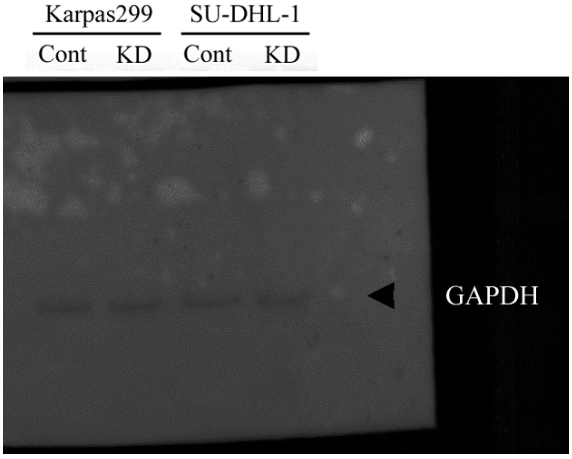

Supplement: Supplementary file 2 — Figure 2 suppl dataset. [file 41598_2020_59530_MOESM2_ESM.pdf]
